# The Jefferson Medical College and the International Medical Congress—Letter from Dr. Roberts Bartholow, Dean

**Published:** 1886-09

**Authors:** Roberts Bartholow

**Affiliations:** Dean of the Jefferson Medical College of Philadelphia


### CORRESPONDENCE.

### THE JEFFERSON MEDICAL COLLEGE OF PHILADELPHIA AND THE INTERNATIONAL MEDICAL CONGRESS.

*Letter from the Dean.*

To the Editor of Daniel's Texas Medical Journal:

I FIND in the June number of your spirited and enterprising JOURNAL, an editorial on Jefferson Medical College, which contains some inaccuracies calculated to do the Institution injury, and which I trust your sense of fairness will permit me to correct.

As respects the International Medical Congress, Jefferson College can have only the interest felt by every loyal American physician, that the meeting in Washington will be a success in every way. The members of the faculty called on to take part, acted as individuals. To prove this, allow me to state a bit of history. I was invited to a place in the organization as made by the original committee, of whom my colleague, Prof Da Costa, was one, but declined the appointment immediately, and before the names were announced. I may say now, my chief reason was, that I had a confident anticipation the arrangement made would prove unsatisfactory, because the organization did not have a sufficiently National character, in that it did not include many of the eminent men of the West and South. The committee having acted, and the organization published to the world, it seemed to me best, for the sake of harmony, to permit the work of the committee to remain undisturbed. Those who agitated the matter at the New Orleans meeting of the American Medical Association, are responsible for the discord that has since occurred, but in this agitation Jefferson Medical College and its faculty have in no way participated. As respects any changes in the instructors of the college, it is quite impossible that the differences in the International Medical Congress could have had any influence; for some members of the faculty had one way of thinking about it, and some another way. Is it not possible for you to believe that the faculty, in the few changes which had been made, had in view only the improvement of the courses of instruction? You are, also, entirely misled as to the position of the College on the "New Code Question." The faculty, and I believe almost the whole of the medical profession of Philadelphia, are firm in their adherence to the Code of the American Medical Association. Therefore, those who have alleged the code question as a reason for our action in any case, simply assert an untruth.

It is an equally erroneous statement that the faculty of the Jefferson Medical College have signalized their opposition to the American Medical Association by uniting in the organization of a new national association. Here, again, each member of the faculty has acted for himself. Several have joined the new body, for purely scientific reasons; several have declined to enter it, lest their purpose be misunderstood, and further cause of discord arise. I may, surely, speak for myself, and especially on points capable of immediate proof. I was invited to unite with other gentlemen in forming the new society, but declined, for the reason that this movement would be construed to mean an organized opposition to the American Medical Association. It has happened as I predicted, and yet I am confident that the gentlemen concerned in the formation of the new society, are governed solely by scientific zeal, and have no sympathy with the new code, and are indifferent to geographical boundaries.

The information given you, in regard to some changes in the corps of Instructors at Jefferson College, is mixed up with so many errors, as to be very misleading. Allow me to state the affair as it really is. If you will kindly refer to the Annual Announcement for 1886, and to those for several years past, you will see that the Instructors continue very much the same. The changes to which you refer took place before "the controversy" in which "the Agnew-Roberts-Hays faction in the International Congress" participated, "beginning with Shoemaker." The change simply consisted in giving up the few lectures of the spring and fall terms, delivered by the gentlemen to whom you refer, because we decided to confine the instruction to our regular corps. Our students were permitted—even advised—to avail themselves of the clinical instruction given by these gentlemen at any of the dispensaries with which they were connected. An attempt has been made, also, to associate the closing of a Post Graduate Course, with these various disturbing questions, as if the college were trying to injure these self-appointed champions of the American Medical Association. Their grievances have been told in the secular press of Philadelphia, where we learned for the first time how our action was viewed by these gentlemen. The truth simply is, that the Post-Graduate course which we had organized, could not be successfully conducted alongside of the under-graduate method of instruction, and we abandoned the field to an institution devoted to that special purpose. The University of Pennsylvania had a similar experience, and their and our post-graduate courses ceased at the same time. That such an incident can be perverted to the purpose of these code agitators, is difficult to realize.

As regards the part which Dr. Pancoast's resignation, and his acceptance of a place in the Medico-Chirurgical College, together with "the excellent gentlemen," who are "all of the Shoemaker-Atkinson politics," it is not easy to speak seriously. If they wish to build up a new College, with any sort of fashionable programme, by all means let them do it. What is that to Jefferson College? Why do they find it necessary to abuse that institution in their attempts to found a new one? We have not opposed them in any way. If they can demonstrate their right to exist by succeeding, we will be entirely pleased. Their success will inure to our benefit. When there was a third Medical College in Philadelphia, Jefferson had never been more prosperous. This has been the uniform experience.

When Bellevue Hospital Medical College entered on its prosperous career in New York, the College of Physicians and Surgeons, and the University Medical College, were also benefitted, and grew apace with the new impetus given to medical teaching. In view of these facts, all that we can desire is to see the new college prosperous; but it must be faithful to its professions, and not merely seek success by exciting animosities and inflaming prejudices.

Very respectfully,

ROBERTS BARTHOLOW, M. D.,

Dean of the Jefferson Medical College of Philadelphia.

